# The impact of blue-green infrastructure on trace contaminants: A catchment-wide assessment

**DOI:** 10.1016/j.wroa.2024.100261

**Published:** 2024-09-27

**Authors:** Marisa Poggioli, Giovan Battista Cavadini, Zhaozhi Zheng, Mayra Rodriguez, Lena Mutzner

**Affiliations:** a Eawag, Swiss Federal Institute of Aquatic Science and Technology, 8600, Dübendorf, Switzerland; b Institute of Civil, Environmental and Geomatic Engineering, ETH Zürich, 8093, Zurich, Switzerland; c School of Civil and Environmental Engineering, University of New South Wales, Kensington, NSW 2052, Australia; d WaterNSW, Parramatta, NSW2150, Australia

**Keywords:** Micropollutants, Stormwater outlets, Combined sewer, Separate sewer system, Sustainable drainage systems (SuDs), Stormwater management model (SWMM), Urban drainage

## Abstract

Blue-green infrastructure (BGI) reduce urban combined sewer overflows (CSOs) and stormwater outlets (SWOs). However, most conventional BGI are not designed to remove trace organic contaminants. Little is known about the potential of conventional BGI to improve surface water quality by reducing the discharge of trace organic contaminants. We derived wash-off loads for street runoff (6PPD-q, DPG, and HMMM), construction materials (diuron), and wastewater-derived contaminants (diclofenac) based on measurements in the combined sewer system. Subsequently, the performance of four BGI types (bioretention cells, green roofs, porous pavements, and urban wetlands) to reduce the discharge of trace organic contaminants via SWOs and CSOs was quantified with a hydrodynamic SWMM model. Moreover, the catchment-wide impact of SWOs and CSOs on surface water was assessed using risk quotients. We found that the annually discharged load can be considerably reduced by implementing BGI. Among the studied BGI types, bioretention cells are the most effective, with a load reduction of up to 80% to surface waters, mainly due to a larger suitable implementation area and a substantial stormwater infiltration. BGI implemented in the separate sewer system are more effective in reducing stormwater contaminant loads than BGI in the combined system. The assessment of the risk quotient in the surface water showed that the concentrations during SWO and CSO discharges exceed the acute environmental threshold in the surface water for 6PPD-q, DPG, diuron, and diclofenac during several events. The implementation of BGI reduced the hours of exceeded risk quotient in the surface water by 93% for bioretention cells. These findings underscore the need for a catchment-wide assessment of future BGI implementations to quantify, manage, and mitigate the impacts of urban pollution.

## Highlights

• Trace contaminants in gray and blue-green urban systems quantified.
• Eco-toxicological impact analysis shows potential risk of tire-wear leachates.
• Blue-green infrastructure can reduce load and impacts on surface waters.
• Bioretention cells are most effective in mitigating impacts on surface waters.
• The most effective solution depends on the urban source of the contaminant.

## Graphical abstract

**Figure:**
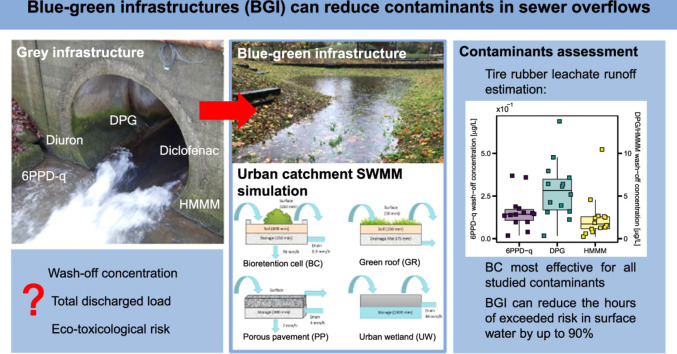

## 1 Introduction

The discharge of urban stormwater and wastewater into surface waters can pose significant eco-toxicological risks (Petrie, 2021; Petrie et al., 2015; Zhang et al., 2024), potentially becoming even more relevant in the future due to an increase in intense rainfall events driven by climate change (Cavadini et al., 2024b; Rodriguez et al., 2024b). Without or with minimal treatment, contaminants can enter surface waters through stormwater discharges in separate systems (SWOs: stormwater outlets) and untreated wastewater mixed with stormwater discharged from the combined sewer systems at the capacity limit (CSOs: combined sewer overflows). Wastewater and stormwater discharged via CSOs and SWOs contain a range of mobile trace organic contaminants (i.e., micropollutants), such as pharmaceuticals, pesticides, and tire-wear leachates washed off urban surfaces (e.g. Gasperi et al., 2012; Mutzner et al., 2020, Mutzner et al., 2016; Spahr et al., 2020; Wicke et al., 2021). Moreover, trace organic contaminants, hereafter called contaminants, can occur in concentrations higher than environmental quality standards, potentially causing adverse environmental impacts on surface water bodies (Mutzner et al., 2022; Spahr et al., 2020).

Alternative urban drainage systems have emerged to address the challenges of urbanization and climate change, relying on blue-green stormwater control measures (Almaaitah et al., 2021). Blue-green infrastructure (BGI) are implemented to retain and partially treat urban stormwater and, thus, can reduce both SWOs and CSOs (Joshi et al., 2021; Kloss, 2012). However, most conventional BGI are not designed to remove mobile contaminants (Rodgers et al., 2022; Spahr et al., 2020; Zhang et al., 2014) and little is known about their potential to improve surface water quality. There are model approaches to estimate the concentrations and risks of contaminants in gray infrastructure systems (i.e., pipes and retention basins) (Brudler et al., 2019; Ianes et al., 2023; Mutzner et al., 2016). However, the water quality improvements in surface waters that can be obtained through a catchment-wide BGI implementation have yet to be quantified.

The goals of this study are to (1) estimate the wash-off concentration from urban surfaces for selected indicator contaminants based on measurements in the combined sewer system, (2) quantify the performance of different BGI types for the load reduction of CSOs and SWOs, and (3) estimate the eco-toxicological impact via risk quotient on surface waters for the status quo and BGI implementations.

Five contaminants were measured in combined sewer systems during wet weather: three from tire rubber leachates (N-(1,3-dimethylbutyl)-N’-phenyl-1,4-benzenediamine-quinone (6PPD-q), 1,3-diphenylguanidine (DPG), and hexamethoxymethylmelamine (HMMM)), one from construction materials (diuron) and a painkiller contained in municipal wastewater (diclofenac). Based on the measurements, the wash-off concentrations from urban surfaces are estimated (6PPD-q, DPG, HMMM, diuron). Four BGI types, bioretention cells (BC), green roofs (GR), porous pavements (PP), and urban wetlands (UW), were simulated with a Storm Water Management Model (SWMM), and SWO and CSO discharges are compared to the status quo gray sewer system (Rodriguez et al., 2024a). The risk quotient in the surface water is quantified for the cumulative load discharged by SWOs and CSOs diluted by the measured flow in the river.

## 2 Results and discussion

### 2.1 Contaminant wash-off concentrations

The wash-off concentration of the studied stormwater contaminants was estimated based on the measured load during wet-weather events in the combined sewer system. The calculations considered the source-specific area contributing to the surface discharge. Diuron was measured at three sites (TWN, IND, OFH) in the study catchment; all other contaminants were measured at one site (TWN) (see Section 4.5).

**Fig. 1:**
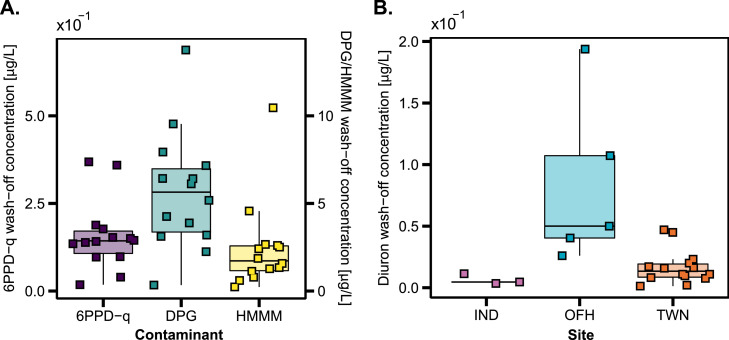
Calculated surface wash-off concentrations based on measured data in the combined sewer system during wet-weather events. **A.** Wash-off concentration from street surfaces for 6PPD-q, DPG, and HMMM **B.** Diuron wash-off concentration from construction materials calculated based on the measured data at three measurement sites: IND = CSO in an industrial area, OFH = One-family houses connected to combined sewer system, TWN = CSO in the town center. Boxplot: The whiskers show 1.5 times the interquantile range, points indicate individual data points, and the solid black line is the median; see geom_boxplot in R (R Development Core Team, 2010). Only the median is shown for site IND, as there are only three data points. Diclofenac shown in Figure SI 1.

The calculated site median concentration (SMC) of tire-rubber leachate 6PPD-q in road surface runoff is 0.14 µg/L (80%-interquantile 0.05 to 0.31 µg/L, Fig. 1A). We calculated lower concentrations of 6PPD-q than in recent studies, where a range of 0.8 to 19 µg/L was reported in stormwater (Saifur and Gardner, 2021; Tian et al., 2021). These previous studies examined runoff from multilane roads, which have considerably higher traffic volumes than those in the studied urban catchment. We estimated DPG median concentrations in road runoff of 5.65 µg/L (80%-interquantile 2.51 to 9.06 µg/L) which is higher than the values reported in the literature of 1.8 µg/L (Saifur and Gardner, 2021), and between 1 µg/L and 3 µg/L (Sandré et al., 2022). This could imply that an additional urban source than road runoff contributes to the measured DPG concentrations. The SMC of HMMM is 1.72 µg/L (80%-interquantile 0.65 to 3.99 µg/L), which agrees well with previously reported data, where an average concentration of 2.32 µg/L with a minimum of 0.88 µg/L and a maximum of 3.89 µg/L was reported (Tamis et al., 2021).

Diuron is commonly added to exterior paints and is expected to leach from plastered walls (Wicke et al., 2021). Thus, diuron is assumed to wash off exclusively from building walls, meaning that the green roof scenario does not reduce the wash-off concentration (Section 2.2). In the model, we distributed the measured diuron load according to the rooftop area, as data on the wall area of the buildings is not available. The measurement site in the one-family house area (OFH) has the highest SMC (0.05 µg/L), followed by the town center (TWN: 0.013 µg/L), and by the industrial site (IND: 0.005 µg/L) (Fig. 1B). The calculated wash-off concentrations at the OFH site are more variable, even though fewer events were sampled than for the TWN site. The higher and more variable wash-off concentrations may originate from recently constructed buildings. For instance, the catchment draining to OFH includes buildings constructed after 2016. In a similar catchment in Berlin, the mean diuron concentration was 0.089 µg/L (Wicke et al., 2021), which is close to the mean of the OFH site of 0.08 µg/L. Also, the mean concentration of 0.017 µg/L at the TWN site is close to the value for the commercial catchment area type in Berlin (mean of 0.026 µg/L) (Wicke et al., 2021). On the other hand, we found lower diuron concentrations at the IND site, which we attribute to the lack of exterior paint on the industrial buildings.

The measured concentrations in wastewater at the site TWN (SMC: 1.81 µg/L, 80%-interquantile 0.40 to 3.71 µg/L) were transformed into load per capita and day (mg/cap/d, Section 4.5 and Figure SI 1) for diclofenac. Previous studies report a load per capita and day of 0.28 ± 0.11 mg/cap/d (Germany) (Letzel et al., 2009) and 0.42–0.67 mg/cap/d (Switzerland) (Tauxe-Wuersch et al., 2005). The per capita concentrations estimated at TWN of 0.54 mg/cap/d are within the range of the Swiss results, whereas the German results are lower but still lay within the estimated range.

Based on measured sewer data, our estimated contaminant concentrations for wash-off from road runoff and construction materials are in a reasonable range, allowing the incorporation of site-specific contaminant wash-off concentrations in urban sewer simulations.

### 2.2 Effect of blue-green infrastructure on discharged contaminant loads

We analyzed the annual discharged contaminant load to assess how different BGI types (bioretention cells (BC), green roofs (GR), porous pavements (PP), and urban wetlands (UW)) reduce the load discharged via CSOs and SWOs into the surface water. The contaminants 6PPD-q, diuron, and diclofenac are discussed below, while DPG and HMMM are shown in SI Section 2 as the discharged load results are comparable to 6PPD-q.

For stormwater contaminants, SWOs are the main substance pathway. WWTPs are responsible for continuous discharges of wastewater contaminants such as diclofenac, while the CSOs and SWOs are responsible for recurring shock loads during rain events. In the study area, SWOs contribute 22% more to the total discharge (in m^3^ a^-1^) than CSOs since they overflow during each rain event. CSOs occur only during events where the combined sewer system is at capacity limit.

**Fig. 2:**
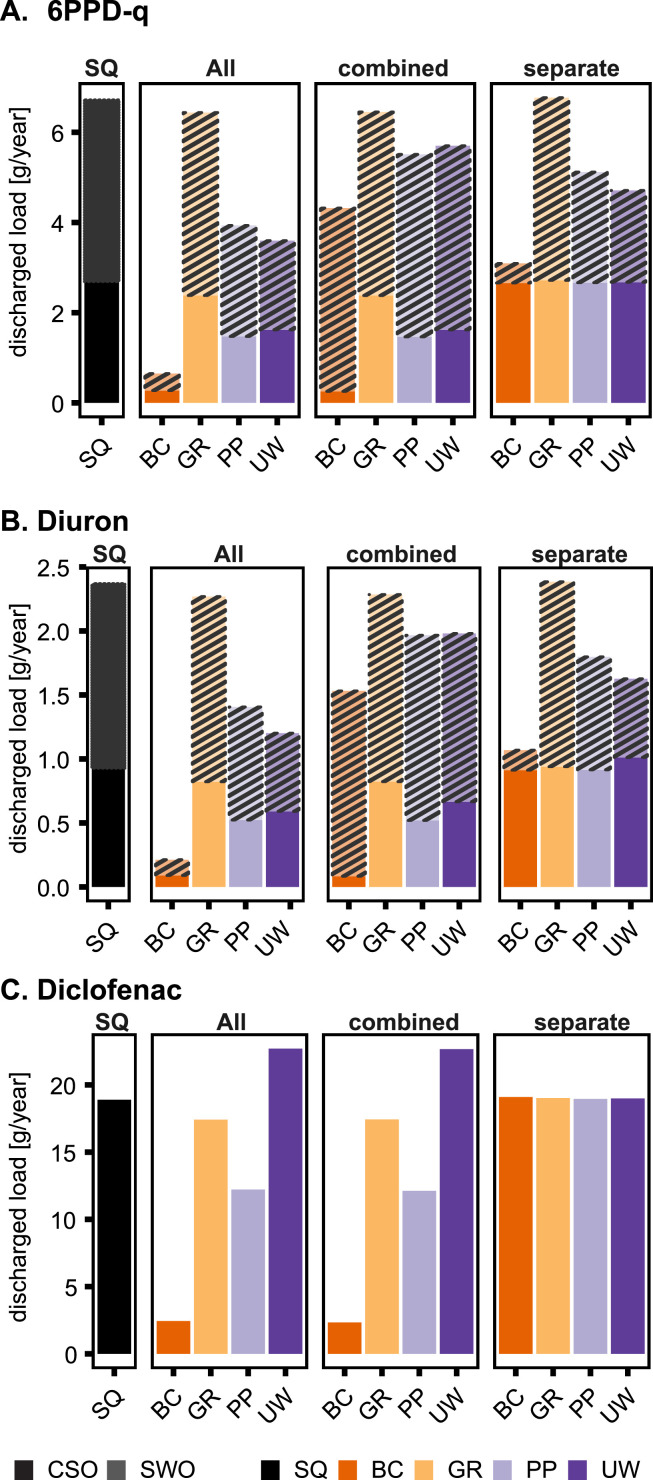
The total load of **A.** 6PPD-q, **B.** diuron, **C.** diclofenac over one year, in gram per year, for all scenarios. SQ: Status Quo; All: BGI in all sub-catchments; combined: BGI only in combined system; separate: BGI only in separate system. The colors represent the BGI implementation types. Results for DPG and HMMM are in Figure SI 2. The hatched area refers to SWOs (top bar), and the ‘normal’ colored area to CSOs (bottom bar).

For stormwater-derived contaminants 6PPD-q and diuron, SWOs contribute 20% more to the total discharged load than CSOs in the status quo (SQ) (Fig. 2A, B). The load reduction is, therefore, higher if BGI are built in the separate system than in the combined system. The contaminant load and removal of GR are assumed to be zero, as the studied contaminants do not occur on GR. Diuron is mainly used in exterior paints on walls and in the model not directly influenced by GR. The only impact of GR is retention and delay of stormwater runoff, thus, a slight reduction of CSO load discharges. Moreover, the suitable implementation area in the separate system is relatively small (Table 1). Thus, GR do not reduce the discharged load of SWOs. The results show a slight increase of 0.7% 6PPD-q load and 0.6% diuron load for GR implemented in the separate system, which we attribute to the uncertainty and continuity error of the model (in the range of −1.04%). Diclofenac enters the aquatic system only via CSOs as we assumed that diclofenac occurs only in municipal wastewater (Fig. 2C). Illicit connections to stormwater systems were not taken into account. Therefore, the BGI implemented in “All” catchments and the “combined system” have the same discharged load, while the scenario “separate system” does not reduce the diclofenac load.

The scenarios with BC show the largest load reduction (over 90%) for all stormwater contaminants. PP and UW are very similar when it comes to the reduction of the total discharged load. PP implemented in the combined system reduce the discharged load more than UW (−18% and −15%, respectively), while for the scenarios in all sub-catchments (All) and separate systems, UW reduce the discharged load slightly more. The difference in discharged load can be attributed to several factors, such as storage capacity and removal rates, with PP demonstrating greater effectiveness in the combined system due to the ability of infiltration in the groundwater (Cavadini et al., 2024a), despite having a lower storage capacity and a smaller area (Table 1).

BC reduce the diclofenac load the most (−87%), followed by PP (−35%) and GR (−8%). The main difference between BC and PP is the larger area and infiltration capacity of BC, thus leading to lower overflow volume (Figure SI 6) and lower loads. GR and UW, on the other hand, have no infiltration, and the load reduction is only based on the retention time of the stormwater volumes, evapotranspiration (in the case of GR), or removal rate (in the case of UW). UW are assumed to be implemented on existing pervious surfaces, thus reducing the imperviousness of the sub-catchment. The stormwater collection of UW leads to smaller CSO peak flow but prolonged outflow into the combined sewer system, causing longer CSO events (Figure SI 7) (Cavadini et al., 2024a). This design can cause an increase in the diclofenac load by 20%. Improving the modeling of UW, such as incorporating evapotranspiration, using a storage node instead of a rain barrel, and connecting UW to the separate sewer system instead of the combined system, would improve the reduction of wastewater contaminants.

No notable differences were found in the BGI load reduction for different stormwater contaminants (SI Section 2). For instance, in the scenario “UW-All,” the total discharged load of 6PPD-q is 3% higher than the diuron load, and for the other BGI types, the difference is below 1% despite different removal rates (Table SI 2). For example, PP has a removal rate of 40% for 6PPD-q and 10% for diuron. However, the 6PPD-q load reduction with PP is less than 1% higher than for diuron, showing that the removal rate does not significantly impact the total discharged load, as discussed in more detail in Section 2.4 and SI Section 6.

The study outcome shows that BC is the most effective BGI type in total load reduction for stormwater and wastewater contaminants, mainly due to the larger suitable implementation area in the catchment, the storage capacity, and the infiltration in the groundwater table. Due to the assumed design, UW are mainly effective when implemented and connected to the separate system, reducing stormwater contaminants loads. Due to the infiltration in the groundwater table, PP show similar results for separate and combined sewer systems, offering a notable load reduction for stormwater and wastewater contaminants despite treating only direct rainfall and having a smaller storage layer. On the other hand, GR is generally ineffective for reducing contaminants load due to a smaller storage capacity and small implementation area. Our study demonstrates that various BGI types and designs significantly influence urban hydrological processes, resulting in substantial differences in load reduction. The influence of BGI parameters is further examined in a sensitivity analysis (Section 2.4). In conclusion, careful BGI design and a catchment-wide assessment of the load reduction are needed to identify the most effective solutions.

### 2.3 Eco-toxicological risk versus load reduction

In the status quo, the stormwater contaminants 6PPD-q, DPG, and diuron reach eco-toxicological relevant concentrations in the receiving surface water due to SWO and CSO discharges (Figure SI 3A-D). In the status quo, the calculated maximum acute risk quotient (aRQ) for 6PPD-q is exceeded in 77% of all events and 615 h in the assessed year. For DPG and diuron, the maximum aRQ is exceeded in 8% (8.2 h per year) and 2% (1 hour per year) of all events, respectively. The hours of exceeded aRQ are reduced the most when BGI are applied in “All” sub-catchments, followed by the separate system for all stormwater contaminants. BC implemented in all sub-catchments reduce the hours of aRQ exceedance the most: 6PPD-q: −93%, DPG: −88%, diuron: −83%, followed by UW with 6PPD-q: −21%, DPG: −71%, diuron: −100%. Diclofenac exceeds aRQ thresholds in 9% of the events and during almost 3 h of the year (Figure SI 3E). The scenarios with BGI in all sub-catchments and the combined system are similar in reducing the hours of exceeded aRQ. BC in the combined system reduces aRQ by −76%. UW in the combined system is the second most effective BGI with a −41% reduction in hours aRQ>1, followed by PP and GR with a reduction of −24% and −12%, respectively.

**Fig. 3:**
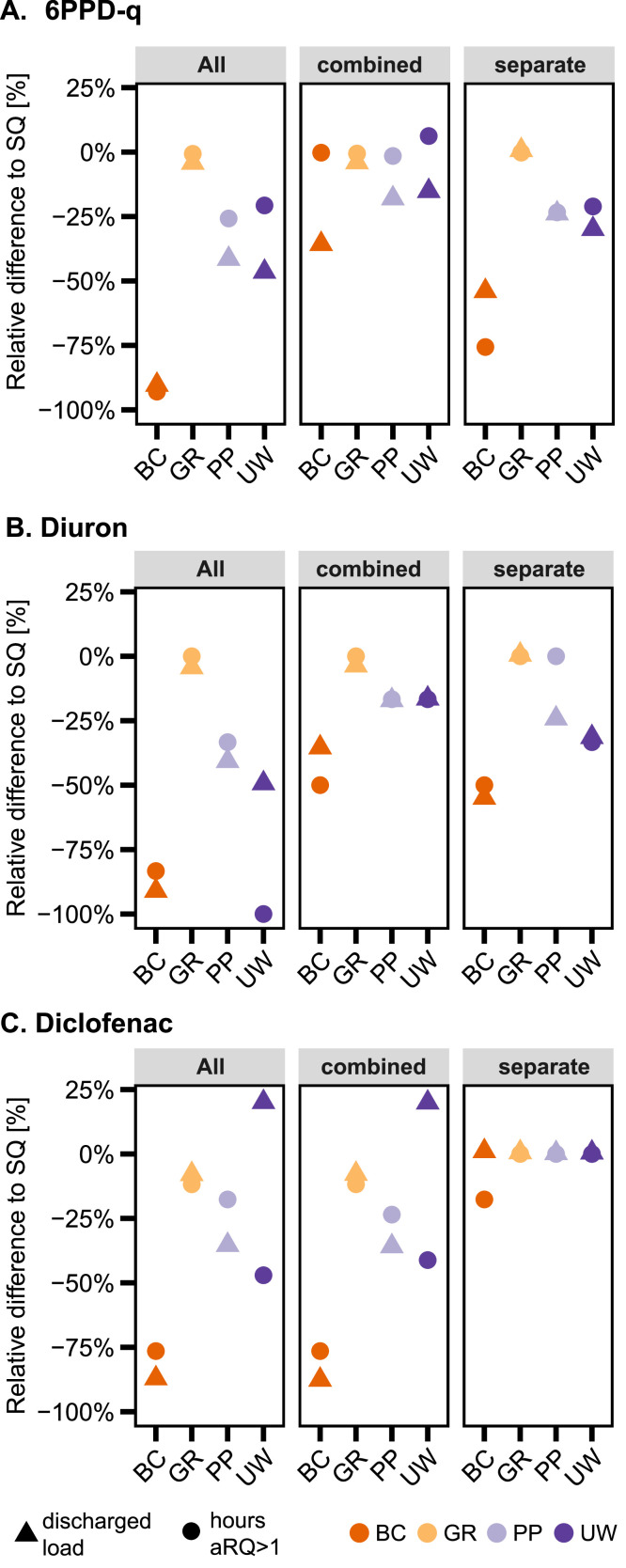
Relative difference to status quo (SQ) of total discharged load and hours of aRQ>1 in [%] for **A.** 6PPD-q, **B.** diuron and **C**. diclofenac. The implemented BGI are bioretention cells (BC), green roofs (GR), porous pavements (PP), and urban wetlands (UW). All: BGI in all sub-catchments; combined: BGI only in combined system; separate: BGI only in separate system. Results for HMMM and DPG are in Figure SI 6.

BC is the most performant BGI type for all contaminants when comparing the hours of exceeding aRQ versus the reduction in discharged load (Fig. 3). On the other hand, GR do not considerably decrease the discharged load and impacts on the receiving water. In the case of diclofenac and diuron, UW reduce the hours of exceedance of aRQ more than the load, as the discharge duration is prolonged but not considerably reduced. For example, in the scenario “All” for diuron the hours of exceeding aRQ is reduced by 100% and the discharged load by 49%. In comparison, PP reduce the hours of exceedance less than the total discharged load, for instance in the scenario “All” for diuron: −33% hours of aRQ and −41% load. The infiltration rate of PP helps to reduce the hours of exceeded aRQ but not sufficiently to avoid CSO and SWO discharges.

We conclude that, depending on the BGI and contaminant type, reducing the discharged loads does not necessarily result in a lower risk to surface waters. Depending on the contaminant, the aRQ can be reduced, although the discharge increases (diuron, UW), while sometimes the opposite happens (6PPD-q, UW). Our results highlight that analyzing the exceedance of the eco-toxicological threshold in the receiving water is essential, as it pinpoints potential risks and effective mitigation solutions from the aquatic environment perspective.

### 2.4 Sensitivity analysis

**Fig. 4:**
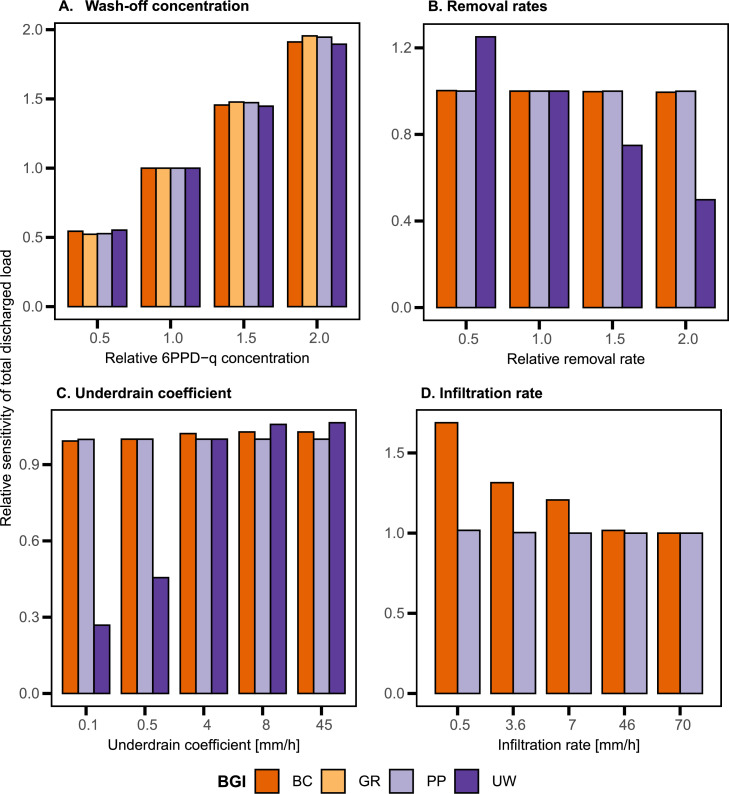
Sensitivity analysis of four input parameters **A.** wash-off concentration, **B.** removal rate, **C.** underdrain coefficient, and **D.** infiltration rate for the total discharged load of the contaminant 6PPD-q in different BGI (bioretention cells (BC), green roofs (GR), porous pavements (PP) and urban wetlands (UW)).

A sensitivity analysis was done to assess the influence of relevant design parameters (Fig. 4 and SI section 6). The washed-off contaminant concentration linearly influences the discharged load for all BGI (Fig. 4A). The removal rate does not influence the discharged load (Fig. 4B), except in the case of UW. For BC and PP, the primary removal of the discharged contaminant load is due to infiltration to the groundwater (Tables SI 4 and SI 5). Changing the infiltration rate of BC has a bigger effect on the discharged load than changing it for PP (Fig. 4D). BC was implemented with a large infiltration rate of 70 mm/h to ensure that most water is infiltrated since it needs to infiltrate the stormwater from the connected impervious areas. Decreasing the infiltration coefficient to the 25-quantile of 0.5 mm/h used in other studies (Hörnschemeyer et al., 2023) causes an increase in the discharged load of 1.7 times. Since the underdrain is not changed, more stormwater is leaving via underdrain, causing longer SWOs (Table SI 4). Lowering the infiltration rate of PP to the 25-quantile (0.5 mm/h) leads to an increase in the load of less than 0.02 compared to the implemented infiltration rate of 7 mm/h. Decreasing the infiltration rate for PP has almost no effect on the discharged load, as the infiltrated stormwater amount does not change significantly since PP only treat direct rainfall. Thus, an infiltration rate of 3.6 mm h^-1^ is sufficient to infiltrate most of the stormwater (Table SI 5).

The removal rate and underdrain coefficient for UW are more sensitive than for other BGI (Fig. 4B and C) due to the larger storage volume. The underdrain coefficient is the most relevant parameter for the discharged load for UW. Decreasing the drain coefficient to the minimum value of 0.5 mm/h (Hörnschemeyer et al., 2023) reduces the discharged load by over −50%. This means that the UW storage capacity chosen in this study is sufficient to store even more water volumes. Changing the underdrain of UW to the 25- and 75-quantile (8 and 45 mm/h, (Hörnschemeyer et al., 2023)) increases the discharged load. GR has the highest catchment-wide surface runoff compared to other BGI (PP: 32%, BC: 5%) due to a small storage layer, thus causing overflows.

### 2.5 Limitations and research needs for a catchment-wide assessment

• We selected contaminants for which we have some indication of their main source. However, there may be other sources of these contaminants in the studied catchment, leading to a potential overestimation of the calculated wash-off amount per source. More data on the sources, occurrence, levels, and spatial variability of contaminants on urban surfaces, such as the accumulation on road surfaces, needs to be collected (Mutzner et al., 2022; Zhang et al., 2024).
• The selected contaminants represent only a fraction of urban runoff and wastewater contaminants. Our study does not give a complete risk profile, which requires further research on eco-toxicological risks, environmental quality standards, and measurement data in sewer systems (Jensen et al., 2024). The environmental quality standards for 6PPD-q, DPG, and HMMM are based on PNEC and LC_50_. Thus, new evidence might lead to a different assessment of the risk quotient. The exceedance of the thresholds for surface waters in our study highlights the need to assess the risk quotient due to SWO and CSO discharges.
• We considered the dilution of CSO and SWO discharges in the surface water to analyze the impacts of wet-weather discharges on surface waters. We did not consider other point sources, such as the WWTP and background concentrations in the surface water, which should be included if a holistic, integrated assessment for entire watersheds is aimed. In addition, the eco-toxicological risk depends on the dilution in the surface water, which has to be adapted for each study area.
• The contaminants removal in BGI is based on coarse assumptions, which seems a good enough estimate as the removal rates have limited influence on the discharged load to surface waters for the selected BGI design parameters (Fig. 4) as most of the stormwater is infiltrated. Nevertheless, more information on removal rates and processes is needed to protect groundwater resources. Our modeling approach does not cover the impact of stormwater infiltration on groundwater, and further research on water quality impact is needed (Speijer et al., 2024).
• In addition, there are limitations in water quality modeling with SWMM as, for example, only dilution is considered in BGI, and in-sewer processes are neglected (Baek et al., 2020; EPA, 2016). In general, there is a lack of knowledge about processes in BGI for trace contaminants (Spahr et al., 2020). The separate system model could not be validated, leading to a higher uncertainty in the SWO discharge estimates.

## 3 Conclusions

• We show the insights gained using indicator contaminants to pinpoint the impact of SWO and CSO discharges on surface waters with and without blue-green infrastructure for an entire urban catchment. We find that blue-green infrastructure can reduce the discharged load through SWOs and CSOs, as well as lowering the risk quotient in the receiving surface water.
• The model assessment of the current system shows that contaminant concentrations can exceed eco-toxicological thresholds in the surface water, calling for mitigation action.
• Based on the chosen design parameters, bioretention cells are the most effective BGI type, with over 80% load and risk reduction for surface water for all studied contaminants. Green roofs are the least effective, with a maximum reduction of 12%.
• The most effective solution to reduce impacts on surface waters depends on the substance sources (stormwater vs. wastewater) in combination with the selected BGI location (separate vs. combined system).
• A comprehensive eco-toxicological impact assessment based on models and validated by measurements is recommended to evaluate the effectiveness of changes in the urban system, such as implementing BGI, in reducing loads and mitigating risks to surface waters.
• For countries relying on groundwater as a water supply, a critical point is the potential infiltration of contaminants into the groundwater through BGI. It is important to quantify the expected contaminant concentrations when BGI infiltrate into the groundwater, and to design appropriate treatments to maximize contaminant removal. More data on contaminant occurrence in urban stormwater and BGI removal rates are needed to mitigate potential environmental and health risks.

## 4 Materials and methods

### 4.1 Urban study catchment

**Fig. 5:**
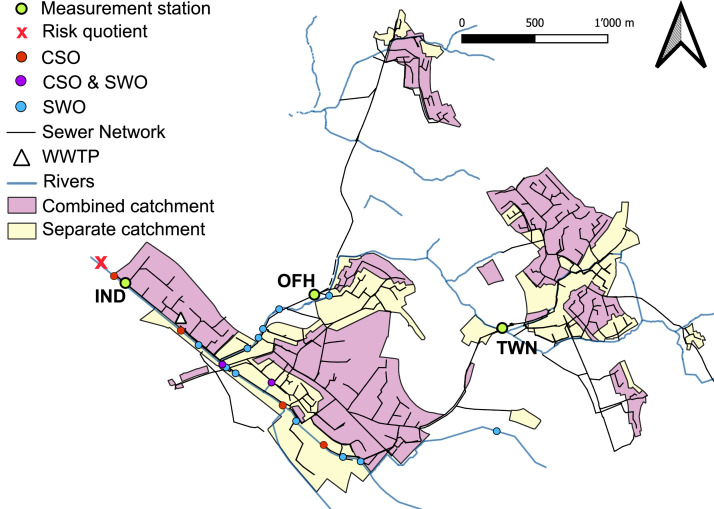
Study area. The purple sub-catchments drain into the combined system and the yellow sub-catchments drain into the separate system. The red dots are CSOs, the purple dots are both SWOs and CSOs and the blue dots are SWOs. The measurement sites are shown on the map with green dots: IND = Industrial area, OFH = One-family houses, and TWN = Town center. The red X indicates the point where the risk quotient was calculated in the surface water.

The urban catchment used for this study is Fehraltorf, a typical Swiss suburban village that is part of the Urban Water Observatory initiative (Blumensaat et al., 2023). The study area is 155 ha, of which 46% are impervious surfaces. Foul sewage from 12,000 people equivalents enters the sewer system and reaches the WWTP. Regarding the sewer system, 61% of the total area drains into the combined system, while the remaining 39% drains into the separate system (Fig. 5). Within the study area, there are a total of 20 CSOs and SWOs, among which four are CSOs. In addition, two overflows are connected to combined and separate systems, acting as both CSOs and SWOs.

### 4.2 Hydrological model and simulations

An existing and calibrated SWMM model of the combined sewer system was extended to assess the potential of BGI implementation on pollutant flows. The original SWMM model covers the combined sewer catchment of Fehraltorf, while the wastewater inflows from the two smaller villages (Russikon and Rumlikon) are monitored through flow measurements and included in the model as boundary inflows (site TWN and OFH in Fig. 5) (Rodriguez et al., 2024a). The model uses Green-Ampt in the infiltration model and the dynamic wave (Rossman, 2015) in the routing model. The rainfall and evaporation time series are obtained from MeteoSwiss, the weather station in Kloten (15 km west of Fehraltorf by MeteoSwiss. CSO outfalls are modeled as SWMM free outfall nodes, with an upstream tank included when applicable (Keller, 2016). The water quality was added to the SWMM model as event mean concentration calculated based on the wash-off of the corresponding source of the contaminant (Section 4.6). The simulations are performed for the year 2019 at a 10-minute timestep, small enough to capture the system's concentration time while having acceptable computation times (Rodriguez, 2022). The calibration was performed using the main combined sewer system trunk flow measurements to represent the overall hydraulic behavior, including CSOs accurately. The model shows good performance, with Nash-Sutcliffe Efficiency (NSE) values above 0.69 for inflows to the wastewater treatment plant (WWTP) (Rodriguez et al., 2024a). A detailed explanation of the original combined sewer system model, the external time series needed for the simulation (rainfall and evaporation), and its calibration are presented in Rodriguez et al. (2024).

However, as contaminants are washed off from urban surfaces and often reach surface waters without treatment, the separate sewer system plays a significant role in contaminant transport. Therefore, we have included the separate system in the SWMM model based on the Cadastral Surveying data (AV Swiss Cadastral Surveying, 2016) and the webGIS of Fehraltorf (webGIS, 2023). Flow measurements for validation are unavailable, leading to higher uncertainty for the flow estimates in the separate system. The separate system representation is considered reliable due to the high accuracy of the cadastral survey data. The SWMM model and data are available at https://doi.org/10.25678/000D5E.

### 4.3 Blue-green infrastructure scenarios

**Fig. 6:**
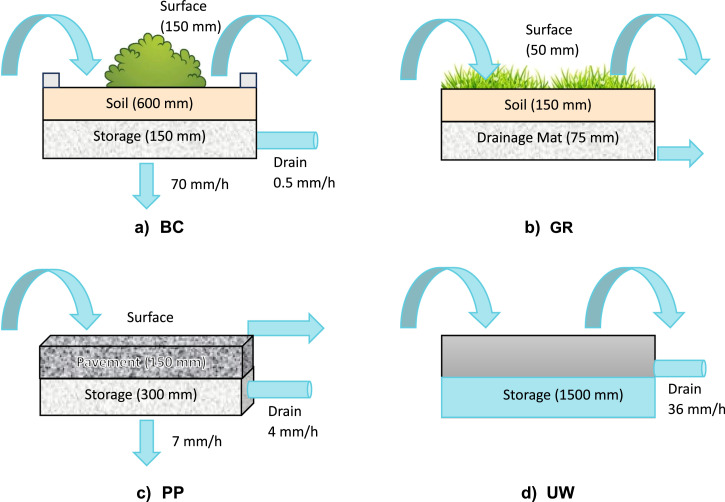
Illustrations of BGI and how they are represented in SWMM. (a) BC: bioretention cell, (b) GR: green roof, (c) PP: porous pavement, and (d) UW: urban wetland (modeled as rain barrel).

Four different BGI were investigated: bioretention cells (BC), green roofs (GR), porous pavements (PP), and urban wetlands (UW) (Fig. 6). These BGI were selected based on different hydraulic treatments of rainwater and implemented in SWMM. The potential suitable area of each BGI in each sub-catchment was determined using land use maps from the municipal cadastral survey, following the approach by Rodriguez et al. (2024a). BC and UW were implemented on previous surfaces such as gardens, green areas, or traffic islands. Thus, they cover the same area. GR is installed on flat roofs, and PP is utilized on impervious surfaces like parking lots or agricultural roads. Since the implementation of GR and PP changes the surface from impervious to pervious, the percentage of imperviousness of the sub-catchment was updated in the SWMM model. We assumed that BGI installations covered 50% of each sub-catchment's potential suitable area (Table 1). Minimum required areas were obtained for each type of BGI (Cavadini et al., 2024a). The BGI design and the included hydrological processes follow existing implementation guidelines and studies (Woods Ballard et al., 2016). GR and PP only treat direct rainfall, while BC and UW collect and infiltrate runoff from impervious areas in the sub-catchment, hence having a larger connected area. BC and PP allow deep percolation, where water can infiltrate the groundwater. UW were implemented as rain barrels, since UW are not included in the “Low Impact Development” tool in SWMM. Rain barrels have a similar structure to UW, as they feature permanent water storage capable of additionally storing stormwater and gradually releasing it. However, rain barrels do not account for evaporation in SWMM, potentially resulting in underestimating the CSO reduction potential. Despite the modeling limitations, rain barrels can serve as a proxy for simulating stormwater storage during rainfall events and offer insights into the potential performance of urban wetlands. The parameters of the BGI are based on existing literature studies (Hörnschemeyer et al., 2023; Joshi et al., 2021; Leimgruber et al., 2018; Rodriguez et al., 2023). The performance of the BGI types is further discussed in Cavadini et al., 2024a. A more detailed description of the different BGI types and their parameters can be found in the SI section 10 (Figure SI 10, Table SI 4).

**Table 1:** Total area of the BGI scenarios (bioretention cells (BC), green roofs (GR), porous pavements (PP) and urban wetlands (UW)), with the respective percentage of the catchment type covarage.

	BC/UW		PP		GR	
Catchment	Area [ha]	Percentage [%]	Area [ha]	Percentage [%]	Area [ha]	Percentage [%]
All	29.5	19	17.6	11.3	5.2	3.3
Combined	17.8	18.8	11.6	12.3	5.1	5.3
Separate	11.6	19.2	6.0	9.8	0.1	0.2

This study analyzed 13 BGI scenarios. The status quo (SQ) scenario represents the absence of BGI structures, while three different approaches for implementing BGI were considered: (1) in all sub-catchments (All), (2) in sub-catchments draining into the combined system, and (3) in sub-catchments draining into the separate system. These different implementation approaches enable us to identify where BGI should be prioritized. The four types of BGI were individually simulated for each approach, with each type implemented on 50% of the suitable area.

### 4.4 Contaminant selection

This study focuses on three contaminant sources: street runoff, construction materials, and municipal wastewater. Tire wear leachates found in street runoff have recently been highlighted due to their toxicity to aquatic organisms (Sieira et al., 2020; Tian et al., 2021). Specifically, the byproduct of N-(1,3-dimethylbutyl)-N’-phenyl-1,4-benzenediamine called 6PPD-quinone (6PPD-q), 1,3-diphenylguanidine (DPG), and hexamethoxymethylmelamine (HMMM), which are assumed to be mainly derived from tire rubber use and wear (Hu et al., 2023), were chosen due to their potential toxicity to aquatic organisms. For instance, the toxicity of 6PPD-q with an LC_50_ of 590 ng/L was identified for brook trout (Brinkmann et al., 2022). Despite the presence of HMMM in water, there are currently no specific water quality guidelines (Rauert et al., 2020). No water quality thresholds (such as EQS) have been established for these three tire wear leaching substances.

Another contaminant of interest is diuron, a herbicide commonly added to exterior paints or coatings of construction materials (Wicke et al., 2021). Studies in the Berlin area have identified diuron concentrations in stormwater runoff as particularly significant (Wicke et al., 2015). Hence, diuron was selected as an indicator contaminant for leachates from construction materials.

In addition, diclofenac, a pharmaceutical compound found in wastewater, was included in the analysis. Diclofenac is commonly found in domestic wastewater (Letzel et al., 2009) and is a useful indicator of municipal wastewater contamination due to its high occurrence and risk to the aquatic environment (Mutzner et al., 2022). Since diclofenac is only present in municipal wastewater , it indicates the CSO pollution reduction when implementing BGI.

### 4.5 Contaminant measurements

Contaminants were measured in three sub-catchments with different urban land uses to cover the variability of contaminant concentrations, illustrated in Fig. 5. The sites were equipped with a discharge measurement device (FloDar 4000SR) and a water level sensor (MB7369 HRXL-MaxSonar-WRM). Composite water sampling was done with automated sampling devices (Sigma 900MAX). The monitoring data was collected between 2016 and 2017 at two measuring points: one at the CSO outfall in an industrial area (IND) and one residential area, where mainly one-family houses were connected (OFH) (Mutzner et al., 2020, Mutzner et al., 2022). At both sites, time-proportional water samples were collected every 5 min and composited over 20 minutes in a glass bottle (Mutzner et al., 2020). Samples were taken at IND when the CSO discharge was active. At OFH, measurements started when a water level of 6 cm was reached in the combined sewer, indicating a significant contribution of stormwater. The third field measurement campaign was conducted between 2021 and 2022 at the inflow of a CSO (Fig. 5, TWN) (Furrer et al., in prep; Furrer et al., 2023). Time-proportional water samples were taken every 2 minutes and composited over 10 minutes (Furrer et al., 2022). In TWN, the measurement started when a water level of 1.8 m was reached in the channel, corresponding to active CSO discharge. All samples were stored at −20 °C until analysis. Table 2 shows the three measurement stations and their sub-catchments used to calculate the contaminant loads per land use area and people equivalents (Section 4.6).

The samples were analyzed using high-performance liquid chromatography coupled with tandem mass spectrometry systems (high resolution and triple quadrupole) using electrospray as an ionization source. The samples were analyzed for several polar organic contaminants; further details on sample preparation and chemical analysis are found in (Furrer et al., in prep; Furrer et al., 2023; Mutzner et al., 2020).

### 4.6 Calculation and simulation of concentrations

The wash-off concentration (C_wash-off_) of each contaminant was calculated based on the measurements during wet weather in the sewer and CSO (Eq. (1)). The median of C_wash-off_ per contaminant, the site median concentration (SMC), was utilized as input for the SWMM model. This concentration was attributed to the relevant land use type based on the estimated primary contaminant source. Contaminants from street runoff are assigned to the street areas, diuron to the building areas, and diclofenac to the people equivalents of each sub-catchment (Table 2). The rain runoff from the source Q_source_ was calculated based on the rainfall and the impervious land use area of the contaminant source for each measured wet-weather event. See SI section 9 for more information.

(1) $$C_{\mathrm{wash}-\mathrm{off}}=\frac{M_{\mathrm{event}}}{Q_{\mathrm{source}}}$$

**Table 2:** Information about the sub-catchments of the measurement sites. IND = CSO in an industrial area, OFH = One-family houses, TWN = CSO in the town center. *A*= area, A_street_ = area of streets in catchment, A_houses_ = area of houses in catchment, *P* = people equivalents.

	A	A_street_		A_houses_		P
	[ha]	[ha]	[%]	[ha]	[%]	[#People]
IND	17.8	0.7	4.1	4.0	22.6	1682
OFH	19.6	2.4	12.3	2.8	14.1	900
TWN	57.7	5.3	9.3	8.5	14.8	3150

C_wash-off_=Wash-off concentration of the contaminant [µg /L]

M_event_=Load at the measurement site during the whole event [µg/event].

Q_source_=Rain runoff coming from the source [L/event^-1^].

For the contaminants 6PPD-q, DPG, and HMMM, measurements from the site TWN were available. Diuron was measured in all three sites. Thus, the SMC for each site was calculated and assigned to sub-catchments with similar land use types (Figure SI 8). The SMC of diclofenac was multiplied by the dry weather flow from each person (300 L/cap/d (Tauxe-Wuersch et al., 2005)) and added as dry weather inflow to the connected nodes of the sub-catchments in the SWMM model.

### 4.7 Removal rates

The contaminant removal rate was added to the BGI with an underdrain in SWMM: BC, PP, and UW (EPA, 2015). No removal rates were used for GR, as we assumed that the selected contaminants do not occur on GR. The removal rate represents the percentage of contaminants removed as water flows through the layers of the BGI (EPA, 2015). The removal rates of contaminants in BGI were based on literature data (Zhang et al., 2023; Zheng et al., 2023). Removal rates of around 40% were found for diuron for constructed wetlands, which we used for UW (Mauffrey et al., 2017; Page et al., 2010, 2011), and 20% for bioretention cells (Fairbairn et al., 2018). No removal rates were found for PP. Hence, we assumed a slightly lower removal rate of 10% than for BC, as PP does not have a soil layer. We did not find removal rates for 6PPD-q, HMMM, and DPG in the literature. Thus, we assumed they have the same removal rates of 50% in BC and 40% in PP and UW. The removal rates are summarized in Table SI 2. Due to the lack of data on the removal rates of BGI, the sensitivity of the removal rates assumptions was analyzed (Section 4.9).

### 4.8 Eco-toxicological risk assessment

The ecological risk to aquatic environments was quantified by calculating the risk quotient (RQ). The concentration in the receiving water was calculated at the catchment outlet (Fig. 5). The loads discharged by all CSOs and SWOs were summed up and divided by the measured river flow in a 10-minute resolution. The upstream concentration in the river was assumed to be zero. Two numerical thresholds in rivers were considered: one for short-term, acute risks (aRQ) (Eq. (2)) and one for long-term, chronic risks (cRQ) (Eq. (3)) (Junghans et al., 2018). The aRQ and the cRQ were calculated for diuron and diclofenac based on environmental quality standards (Ecotoxcentre, 2023). For the substances 6PPD-q, DPG, and HMMM, no environmental quality standards were found for the risk calculation. Therefore, the PNEC was used for HMMM (Slobodnik et al., 2012) and the LC_50_ for 6PPD-q and DPG (Sandré et al., 2022; Tian et al., 2021). The LC_50_ was divided by an assessment factor of 1000 to account for the uncertainty in the toxicity data (von der Ohe et al., 2011). The resulting environmental quality standards are listed in Table SI 1.

(2) $$\mathrm{aRQ}=\;\frac{C_{\mathrm{river}}}{\mathrm{MAC}-\mathrm{EQS}}$$

aRQ=Acute risk quotient [-].

C_rive__r_=Concentration C(t) in the river [µg/L] in 10 min resolution

MAC-EQS=Maximum allowable concentration EQS, Table SI 1 [µg/ L].

(3) $$\mathrm{cRQ}=\;\frac{C_{\mathrm{river},2\mathrm{weeks}}}{\mathrm{AA}-\mathrm{EQS}}$$

cRQ=Chronic risk quotient [-].

C_river,2weeks_=Concentration C(t) in the river averaged over 2 weeks [µg/L]

AA-EQS=Annual average concentration EQS, Table SI 1 [µg/L].

An overflow event was defined as a period with exceeds a contaminant concentration above 0 µg/L showing an active discharge. An overflow event was considered to be finished when no contaminants were detected for at least 6 h.

### 4.9 Sensitivity analysis

Four parameters were tested in the sensitivity analysis: input concentration (SMC), removal rate, underdrain coefficient, and infiltration rate. The input concentration and removal rate were multiplied by factors of 0.5, 1.5, and 2 to assess the sensitivity of the discharged total load with respect to potential concentration and removal rate ranges (Table SI 3). The underdrain coefficient and infiltration rate were varied according to the parameter values reported in Hörnschemeyer et al. (2023). The relative influence of the parameters changes on the annual discharged load via CSOs and SWOs was calculated, where the relative influence was expressed as a ratio of the values obtained in the status quo. A value of 1 represents the value obtained in the status quo, 0.5 represents half, and 2 represents double. All results were computed for 6PPD-q to show the effect of changes in input parameter values.

## Declaration of generative AI and AI-assisted technologies in the writing process

During the preparation of this work, the authors used Grammarly and ChatGPT to check grammar and spelling. After using this tool/service, the authors reviewed and edited the content as needed and take full responsibility for the content of the publication.

## CRediT authorship contribution statement

**Marisa Poggioli:** Writing – review & editing, Writing – original draft, Visualization, Validation, Methodology, Investigation, Formal analysis, Data curation, Conceptualization. **Giovan Battista Cavadini:** Writing – review & editing, Validation, Software, Investigation, Data curation, Conceptualization. **Zhaozhi Zheng:** Writing – review & editing, Data curation. **Mayra Rodriguez:** Writing – review & editing, Validation, Software, Methodology, Investigation, Data curation. **Lena Mutzner:** Writing – review & editing, Validation, Supervision, Software, Resources, Project administration, Methodology, Investigation, Funding acquisition, Data curation, Conceptualization.

## Declaration of competing interest

The authors declare the following financial interests/personal relationships which may be considered as potential competing interests:

Lena Mutzner reports financial support was provided by Swiss National Science Foundation. If there are other authors, they declare that they have no known competing financial interests or personal relationships that could have appeared to influence the work reported in this paper.

## Data Availability

The data is available through Eawag's Research Data Institutional Collection (ERIC-open): https://doi.org/10.25678/000D5E

## Appendix Supplementary materials

Image, application 1
