# Supplementary material for: The impact of blue-green infrastructure on trace contaminants: A catchment-wide assessment
Source: Water Res X. 2024 Sep 27;25:100261. doi: 10.1016/j.wroa.2024.100261 (PMC11488434; doi:10.1016/j.wroa.2024.100261)
Supplement: Supplementary file 1 [file mmc1.docx]

The impact of blue-green infrastructures on trace contaminants: A catchment-wide assessment

Marisa Poggioli^1,2^, Giovan Battista Cavadini^1,2^, Zhaozhi Zheng^3,4^, Mayra Rodriguez^1^, Lena Mutzner^1,*^

*^1^* *Eawag, Swiss Federal Institute of Aquatic Science and Technology, 8600 Dübendorf, Switzerland.*

*^2^ Institute of Civil, Environmental and Geomatic Engineering, ETH Zürich, 8093 Zurich, Switzerland.*

*^3^* *School of Civil and Environmental Engineering, University of New South Wales, Kensington, NSW 2052, Australia*

*^4^ WaterNSW, Parramatta, NSW2150, Australia*

**lena.mutzner@eawag.ch*

**SUPPLEMENTARY INFORMATION**

# Contaminant wash-off concentration


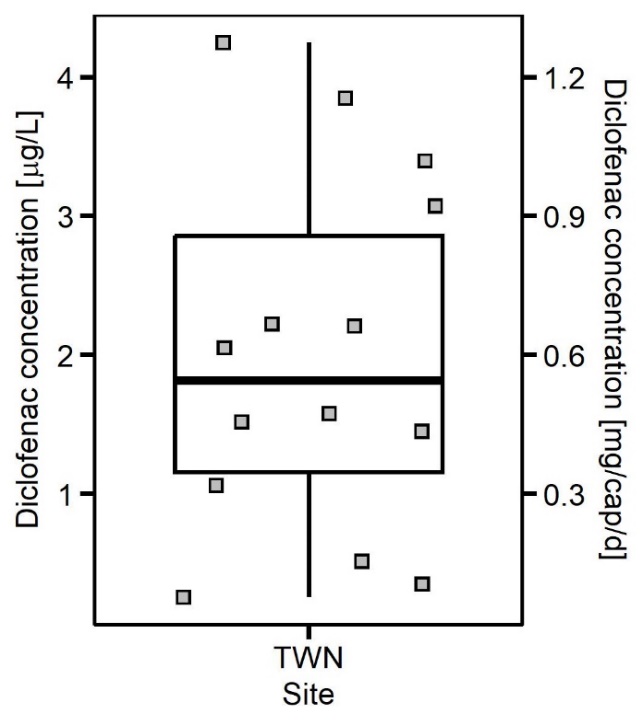


**Figure SI 1.** Estimated concentration of diclofenac in municipal wastewater based on measurements in the combined sewer system at the site TWN. The grey dots represent the single measurements. The secondary y-axis shows the load per capita and day. Boxplot: The whiskers show 1.5 times the interquantile range, points indicate individual data points, and the solid black line is the median, see geom_boxplot in R (R Development Core Team, 2010).

# Effect of blue-green infrastructure on discharged loads

| 1. **DPG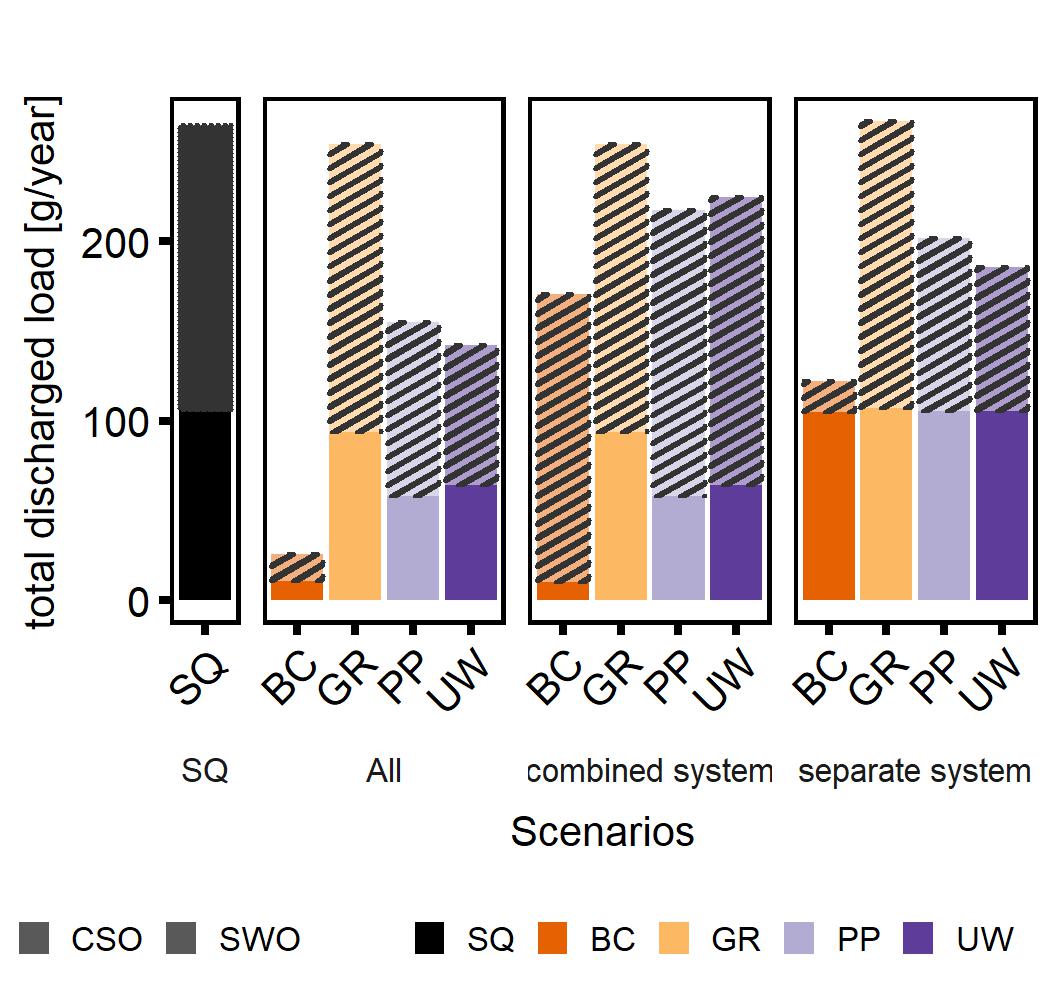** |
| --- |
| 1. **HMMM**   **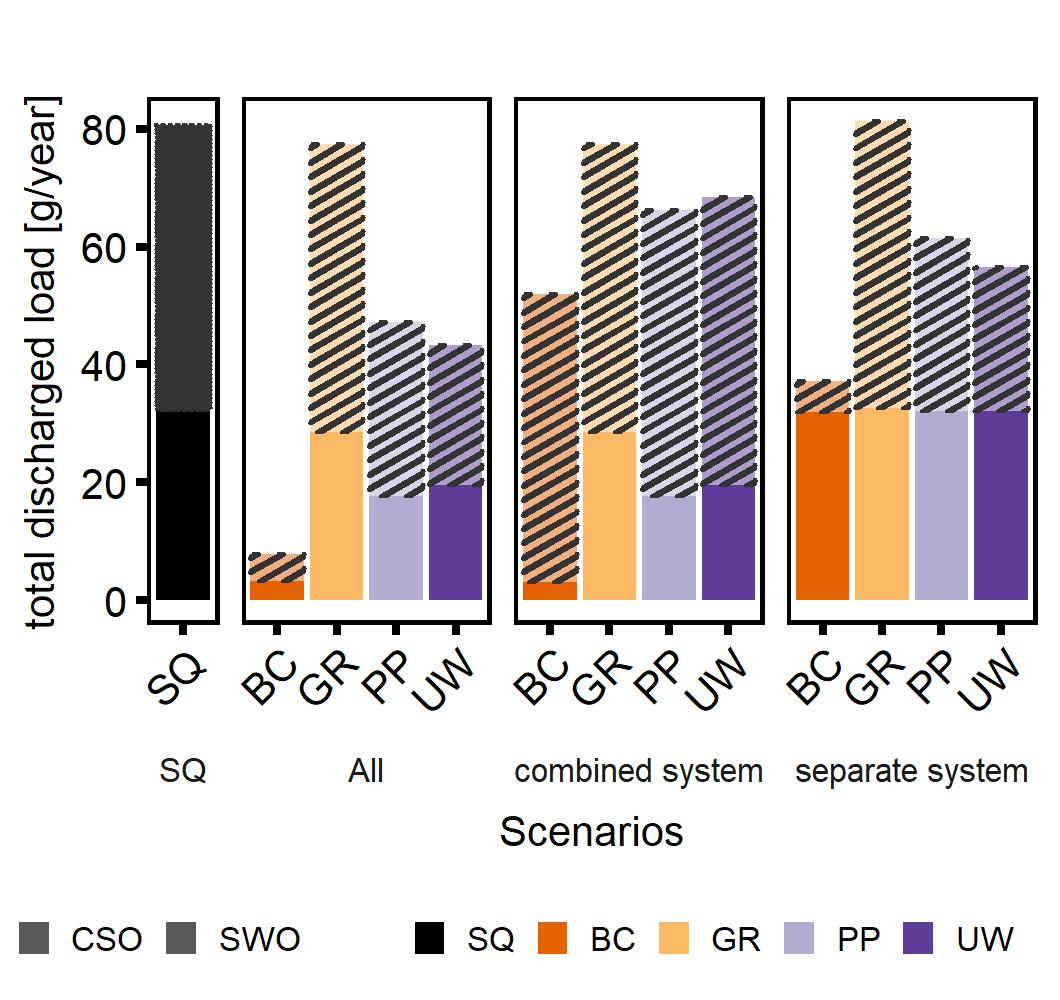** |

**Figure SI 2.** The total discharged load of **A.** DPG and **B.** HMMM over one year for all scenarios. SQ: Status Quo, All: Blue-Green Infrastructure (BGI) in all sub-catchments, combined system: BGI only in combined system, separate system: BGI only in separate system. The hatched area refers to SWO (top bar), and the ‘normal’ coloured area to CSO (bottom bar).

# Eco-toxicological risk in receiving water

The following approach was used to determine the threshold values for surface waters for all investigated substances to determine the acute aquatic eco-toxicity (Tamis et al., 2021):

- 1. If an Environmental Quality Standard (EQS) existed for the substances, these EQS values were given priority.
  2. If no EQS value was available for the particular contaminant, the scientific literature was searched for reported Predicted No Effect Concentration (PNEC) values.
  3. If no PNEC value was found in the literature, an alternative approach was adopted. The LC50 was utilized, applying an assessment factor of 1000 to correct for the uncertainty in the toxicity data and ensure protection from non-specific toxic effects (TGD, 2003; von der Ohe et al., 2011).

The short-term EQS expressed as maximum allowable concentration (MAC-EQS) was used for the acute RQ (Spahr et al., 2020). For the substances 6PPD-q, DPG, and HMMM, no EQS were found. Therefore, the PNEC was used for HMMM (Slobodnik et al., 2012) and the LC_50_ for 6PPD-q and DPG (Sandré et al., 2022; Tian et al., 2021) with an assessment factor of 1000 (Table SI 1). Diuron has a MAC-EQS that is recommended by the Swiss Ecotox Center (Ecotoxcentre, 2023). Diclofenac has no MAC-EQS. Therefore, the ratio of 10 between acute and chronic conditions was used to predict chronic toxicity thresholds using acute toxicity values (Ahlers et al., 2006; Tamis et al., 2021). The long-term EQS expressed as annual average concentration (AA-EQS) is used to evaluate the chronic aquatic eco-toxicity (Spahr et al., 2020). Diuron and diclofenac have an AA-EQS (Ecotoxcentre, 2023); all other contaminants do not.

**Table SI 1.** The MAC-EQS and long-term AA-EQS of the contaminants used to calculate the RQ. (a) Brinkmann et al. (2022), (b) Sandré et al. (2022), (c) Slobodnik et al. (2012), (d) (Ahlers et al., 2006; Tamis et al., 2021), (e) UVEK (Eidgenössisches Departement für Umwelt (2020), (f) Ecotoxcentre (2023)

|  | MAC-EQS  [µg/L] | AA-EQS  [µg/L] |
| --- | --- | --- |
| 6PPD-q | 0.00059 (a) | - |
| DPG | 4.2 (b) | - |
| HMMM | 54 (c) | - |
| Diclofenac | 0.5 (d) | 0.05 (e,f) |
| Diuron | 0.25 (f) | 0.07 (e,f) |

| 1. **6PPD-q**    |  |
| --- | --- |
| 1. **DPG**    | |
| 1. **HMMM**   **** | |

| 1. **Diuron**   **** |
| --- |
| 1. **Diclofenac**   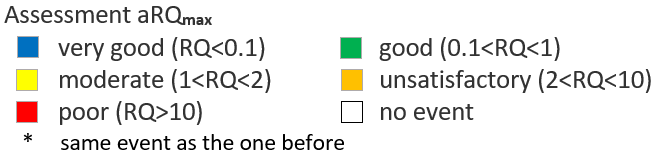  **Figure SI 3.** The maximal acute risk quotient (aRQmax) of **A.** 6PPD-quinone, **B.** DPG, **C.** HMMM, **D.** diuron, **E.** diclofenac for each event of the year 2019 for the different scenarios: SQ: Status Quo, All: BGI in all sub-catchments, CS: BGI only in combined system, SS: BGI only in separate system. The star symbol shows cases where the two events from the status quo merged into one event (event defined as 6 h of no overflow). |

**Stormwater contaminants.** The maximum acute risk quotient (aRQ) of each overflow event is often higher than 10 for 6PPD-q for all of the analyzed scenarios (Figure SI 3A). The results show that 6PPD-q mostly has a poor aRQ, which means that the concentrations are very high compared to the threshold value. One reason could be the low PNEC used for 6PPD-q of 5.9 10^-4^ µg/L reported for brook trout (Brinkmann et al., 2022), which lives in Swiss rivers.

The BGI have varying effects on the eco-toxicological risk due to 6PPD-q discharges. While GR do not affect the aRQ, BC have the greatest impact on the aRQ, transforming 82% of events with aRQ>1 into good or very good events. When BC are implemented in all sub-catchments, the maximum aRQ is reduced the most (7% of time exceeded) compared to the scenario with BC only in the separate system (25% of time exceeded). Generally, BGI in the whole catchment or only in the separate system lead to similar results. Both reduce the aRQ more than the scenarios with BGI only in the combined system. There are four events where the aRQ is increased from good in status quo to aRQ>1 with UW implemented in all sub-catchments. Figure 3 displays the maximum aRQ for each event, indicating the peak concentration corresponding to the maximum risk. UW may increase these peaks due to full storage and the maximum capacity of the drainage system. Thus, a larger volume of water reaches the CSO or SWO than the status quo. We implemented UW in each sub-catchment individually, and the drainage flow and overflow of UW were returned to the combined system. In most cases, UW would instead drain into the separate systems, thus contributing to a higher reduction of 6PPD-q loads via CSO than in our simulation.

The other stormwater contaminants show similar results to 6PPD-q. For DPG, it is visible that the one event with a poor aRQ was only improved by implementing BC and UW in all sub-catchments (Figure SI 3B). The same applies to diuron (Figure SI 3D), where ”BC-All” and “UW-All” reduce the worst event, an unsatisfactory event (2 < aRQ > 10) in the status quo, to a moderate and good event, respectively. The other event with aRQ>1 is not improved by GR but by all other BGI. HMMM never exceeds the aRQ of 1 due to a high acute threshold value (Figure SI 3C).

**Wastewater contaminant.** The maximum aRQ of the wastewater contaminant diclofenac is shown in Figure SI 3C. The ”SS” scenarios do not affect the aRQ of diclofenac. There are two exceptions in the scenario ”BC separate system”, where the aRQ is lowered due to two SWO acting like CSO in the study catchment area. The scenarios with BGI in all sub-catchments or only in the combined system are similar in improving the maximal aRQ or preventing entire CSO events. In both cases, BC reduces the number of events by over -80% and the number of events with aRQ>1 by -60%. PP is the second most effective, with a -33% reduction in events and a -40% reduction in the number of events with aRQ>1. GR prevent 10% of events from happening, but are the least effective. UW, on the other hand, prevent 22% of event from happening, even though they increase the discharged diclofenac load compared to the status quo (see Figure 2). On the other hand, four events in the UW scenario have a higher aRQ than the status quo. For UW, overflow events are longer due to storage; therefore, the peak occurs at another time point in the river, resulting in a higher aRQ due to lower flow in the river (Figure SI 7).

**Comparison of BGI types**. BC and UW reduce the total number of events of the year for stormwater contaminants compared to the status quo (6PPD-q: -16% with “BC-All” and -9% with “UW-All,” diuron: -61% with “BC-All” and -12% with “UW-All”, see Figure SI 3A and D). For stormwater contaminants, the BGI UW reduces the maximum aRQ more effectively (6PPD-q: -15% with “PP-All,” -19% with “UW-All”), but PP reduce the hours of exceeded aRQ more (6PPD-1: 400 hours aRQ>1 with “PP-All”, 426 hours with aRQ>1 with “UW-All”). The results of all contaminants show that BC contribute the most to reduce the maximum aRQ for both stormwater and wastewater contaminants. The chronic risk quotient (cRQ) of diuron and diclofenac is never exceeded and therefore not further discussed (Figure SI 4).

| 1. **Diuron**   **** |
| --- |
| 1. **Diclofenac**   ****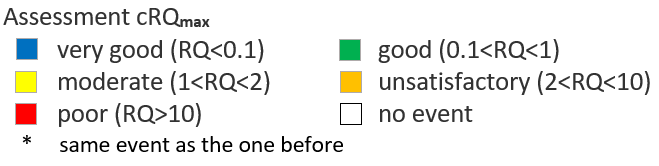 |

**Figure SI 5.** The maximal chronic risk quotient (cRQmax) of **A.** diuron and **B.** diclofenac for each event of the year 2019 for the different scenarios. SQ: Status Quo, All: BGI in all sub-catchments, CS: BGI only in combined system, SS: BGI only in separate system. The star symbol shows cases where the two events from the status quo merged into one event (event defined as 6 h of no overflow).

# Eco-toxicological risk versus load reduction

| 1. **DPG**   **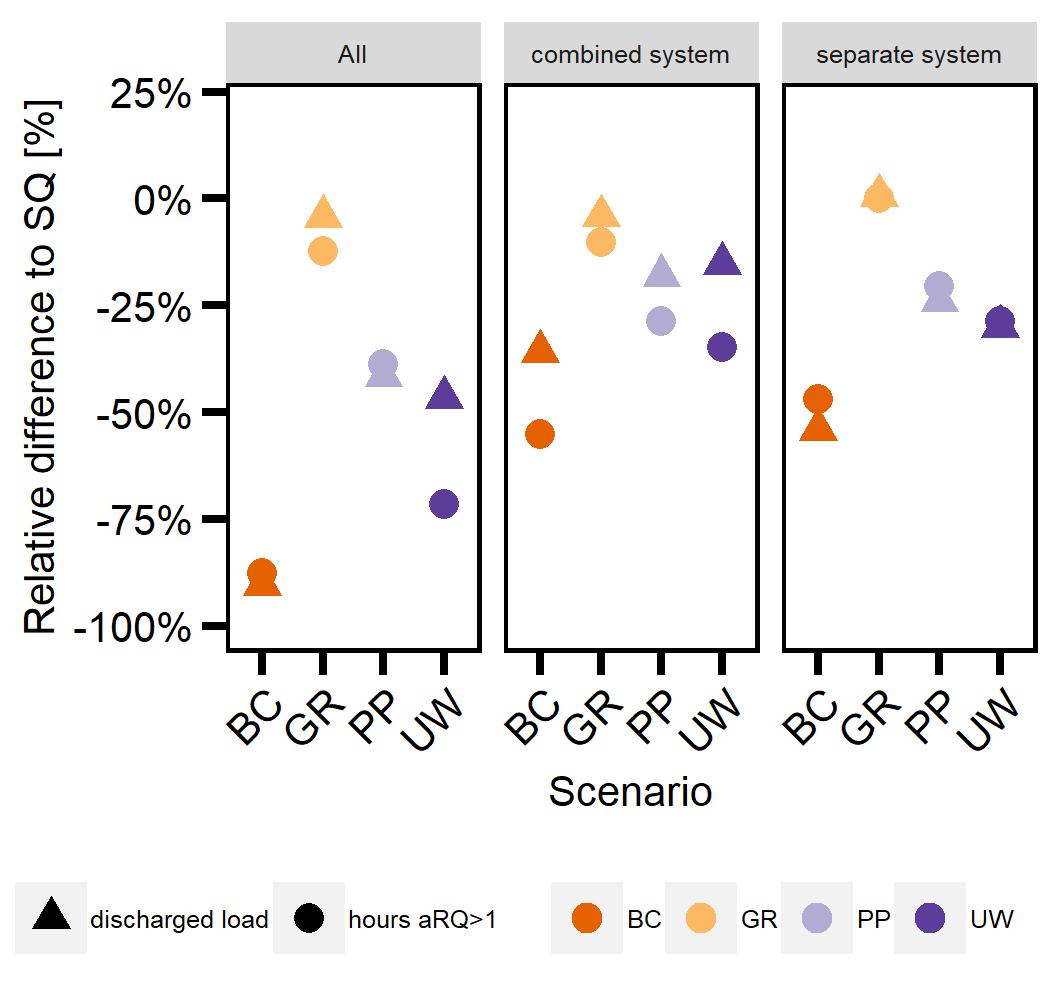** |
| --- |
| 1. **HMMM**   **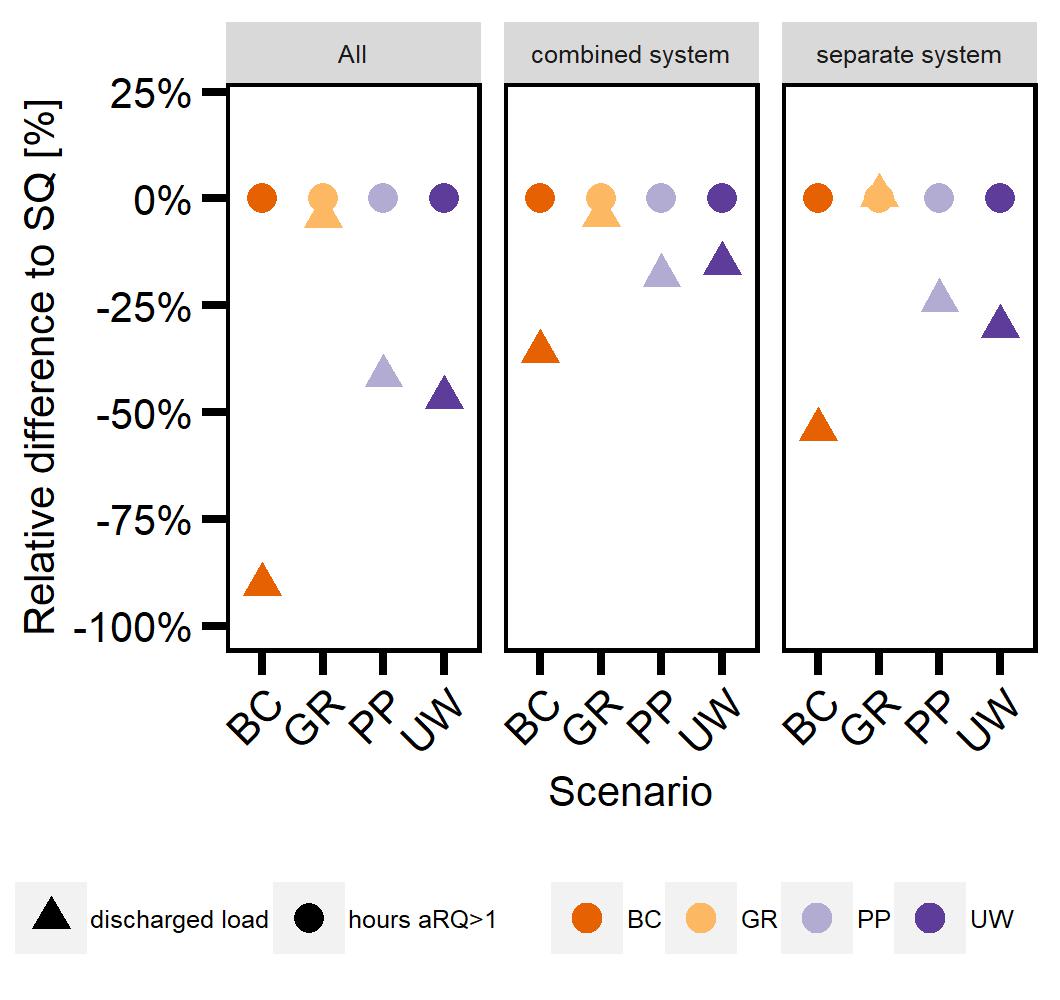** |

**Figure SI 6.** Relative difference to status quo (SQ) of total discharged load and hours of aRQ>1 in [%] for **A.** DPG and **B.** HMMM. The implemented BGI are bioretention cells (BC), green roofs (GR), porous pavements (PP), and urban wetlands (UW). All: BGI in all sub-catchments, combined system: BGI only in combined system, separate system: BGI only in separate system.

# Removal rates

**Table SI 2.** The removal rates of the contaminants in % used in the BGI in SWMM.

|  | BC  [%] | PP  [%] | UW  [%] |
| --- | --- | --- | --- |
| 6PPD-q | 50 | 40 | 40 |
| DPG | 50 | 40 | 40 |
| HMMM | 50 | 40 | 40 |
| Diuron | 20 | 10 | 40 |

# Sensitivity analysis

**Table SI 3.** Parameters used for the sensitivity analysis: Site Mean Concentraiton (SMC) and removal rates of 66PD-q.

|  |  | 0.5 | 1 | 1.5 | 2 |
| --- | --- | --- | --- | --- | --- |
| SMC 6PPD-q | [µg/L] | 0.072 | 0.143 | 0.215 | 0.286 |
| Removal rate BC | [%] | 25 | 50 | 75 | 100 |
| Removal rate PP | [%] | 20 | 40 | 60 | 80 |
| Removal rate UW | [%] | 20 | 40 | 60 | 80 |
|  |  |  |  |  |  |

**Table SI 4**. Infiltration rates used in the sensitivity analysis based on values reported by Hörnschemeyer et al. (2023). Average water balance of a bioretention cell BC installation for the different infiltration rates considered in the sensitivity analysis.

| Infiltration rate [mm/h] | ET loss [%] | Infiltration loss [%] | | | Surface outflow [%] | Drainage outflow [%] | | Final storage [%] |
| --- | --- | --- | --- | --- | --- | --- | --- | --- |
| 0.5 | 11.1 | | 27.9 | 21.5 | | 37.5 | 1.9 | |
| 3.6 | 11.1 | | 46.9 | 19.9 | | 20.3 | 1.8 | |
| 7 | 11.1 | | 54.7 | 18.7 | | 13.7 | 1.8 | |
| 46 | 11.1 | 71.9 | | 12.6 | | 2.6 | 1.8 | |
| 70 | 11.1 | 74.6 | | 10.9 | | 1.6 | 1.8 | |

**Table SI 5.** Infiltration rates used in the sensitivity analysis based on values reported by Hörnschemeyer et al. (2023). Average water balance of a porous pavement installation for the different infiltration rates considered in the sensitivity analysis.

| Infiltration rate [mm/h] | ET loss [%] | Infiltration loss [%] | Surface outflow [%] | Drainage outflow [%] | Final storage [%] |
| --- | --- | --- | --- | --- | --- |
| 0.5 | 12.2 | 85.1 | 0.0 | 2.8 | 0.0 |
| 3.6 | 1.2 | 98.1 | 0.0 | 0.7 | 0.0 |
| 7 | 0.4 | 99.4 | 0.0 | 0.2 | 0.0 |
| 46 | 0.0 | 100.0 | 0.0 | 0.0 | 0.0 |
| 70 | 0.0 | 100.0 | 0.0 | 0.0 | 0.0 |

# Discharged load versus overflow volume


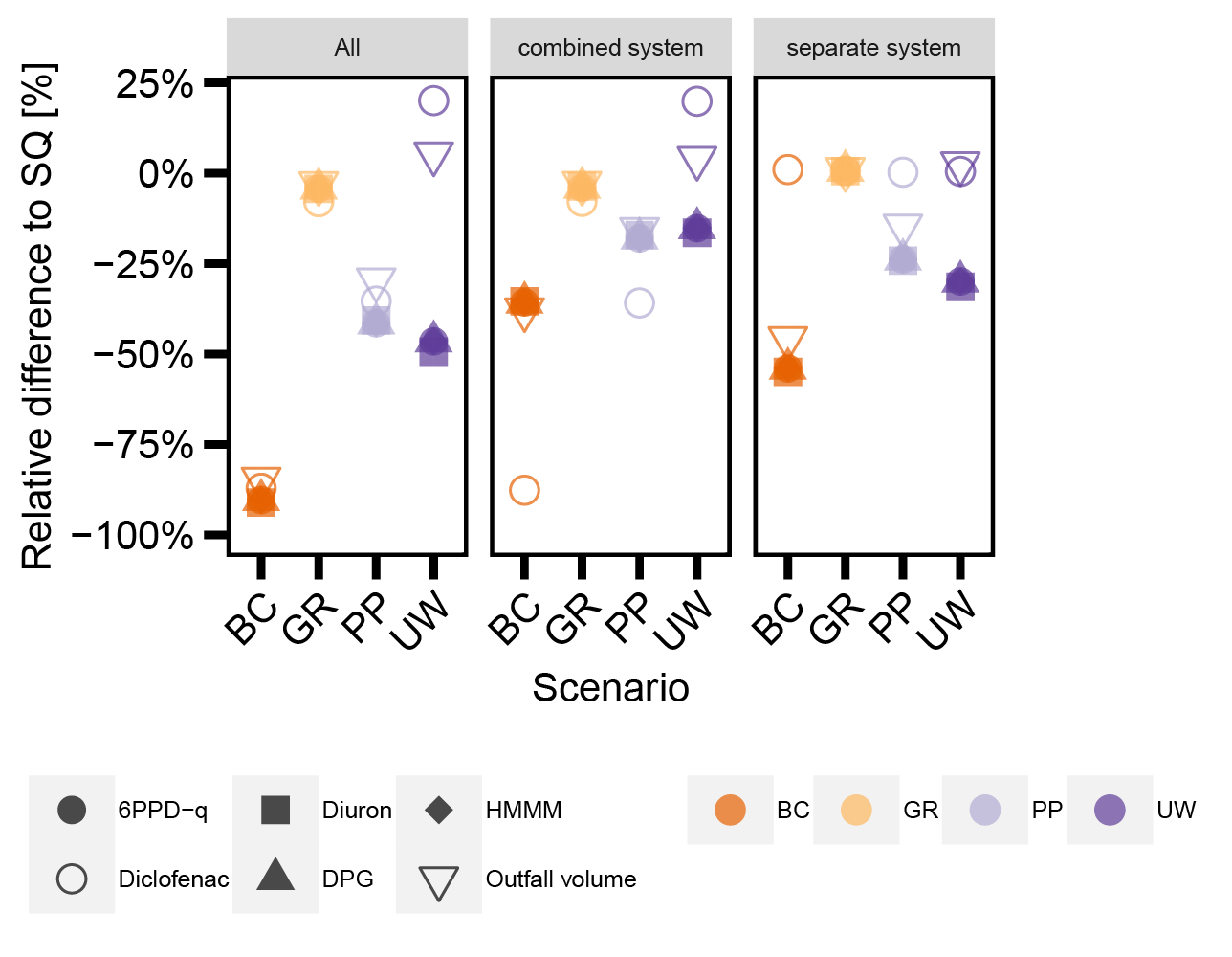


**Figure SI 7.** Relative difference to status quo (SQ) of a total discharged load of 6PPD-q, DPG, HMMM, diuron, and diclofenac compared to the total outfall volume [m^3^/a]. The implemented BGI are bioretention cells (BC), green roofs (GR), porous pavements (PP), and urban wetlands (UW). All: BGI in all sub-catchments, combined system: BGI only in combined system, separate system: BGI only in separate system.

# Discharged load of one event

**
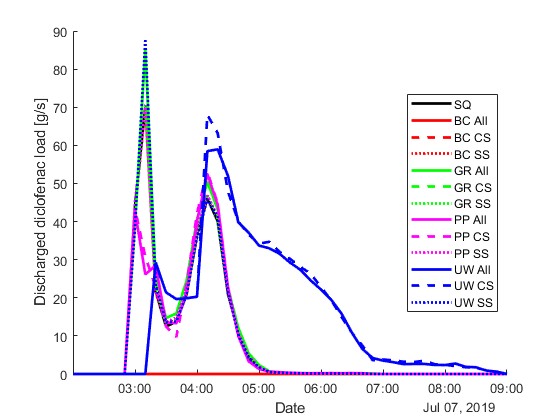
**

**Figure SI 8.** Discharged diclofenac load of the event of 07.07.2019 for the different scenarios.

# Contaminant sources and wash-off

The rainfall during each measured event was summed up to estimate the rain runoff that comes exclusively from the contaminant source of interest (Eq. 1). The start of the event was shifted backward in time by the minutes it takes for the runoff to reach the measurement station if it starts raining. The calculation for the time delay is shown in Eq. 2. This same time delay was used for all measurement stations.

$Q_{source}=R_{event}\cdot\alpha\cdot A_{source}\cdot10000$ (1)

Q_source_ Rain runoff from the contaminant source [L event^-1^]

R_event_ Rainfall over the whole event [mm/event]

α Discharge coefficient of the source (street = 0.8, houses = 1)(Gujer, 2007)

A_source_ Area of the source draining to the measurement site [ha]

$t=\frac{L}{v}=\frac{1300m}{2ms^{-1}}\approx10min$ (2)

t Timeshift for the rainfall [min]

L Length from the point furthest away from the measurement station TWN [m]

V Velocity in the drainage system (Gujer, 2007) [m s−1]

The estimated contaminant concentrations were assigned to the percentage of area of each sub-catchment representing their estimated primary source. Contaminants from street runoff are assigned to the street areas, diuron to the building areas, and diclofenac to the people equivalents of each sub-catchment. Since diuron was measured at all three measurement sites, three different wash-off concentrations were calculated. Land use types were assigned to the different wash-off concentrations of diuron. Residential, Industry, and Commercial land use types were used to categorize the sub-catchments (Joshi et al., 2021). The residential-commercial mix defined by Joshi et al. (2021) was defined as “Center.” Sub-catchments with only paved or green land cover, according to the Cadastral Surveying data (AV Swiss Cadastral Surveying, 2016), were categorized correspondingly. The land use type assigned to each sub-catchment is visible in Figure SI 8. To the land use type Industry, the SMC of the site IND was assigned, since this site covers the industry of Fehraltorf. The residential areas receive the SMC of the site OFH since this site represents a residential estate with one-family houses. Commercial and the Center are more of a mixed land use type with buildings, as well as some industry or public buildings. Therefore, the SMC of the site TWN was assigned since this represents the village Russikon with the city center, houses, and few industries. No SMC was assigned to the land use types Paved and Green since no buildings and, therefore, no diuron concentration is expected.


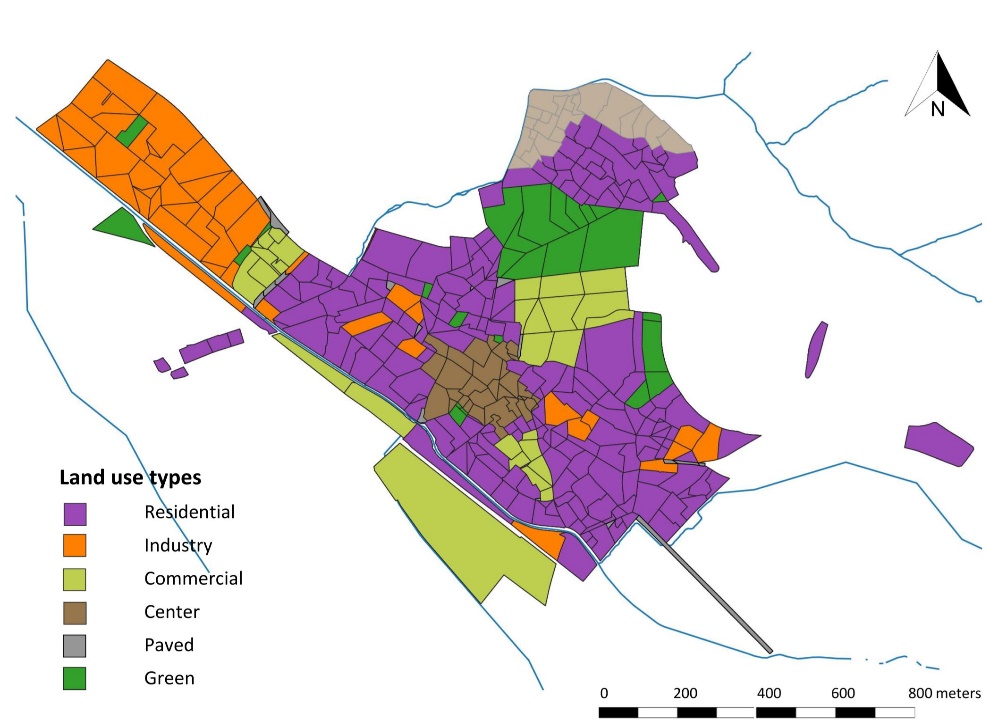


**Figure SI 9.** Land use type is attributed based on detailed land use distribution (see also Figure SI 9 on land use distribution).


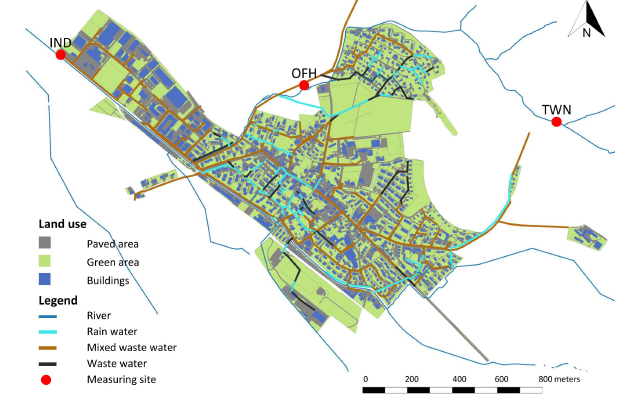


**Figure SI 10.** The land use distributions are visually represented as follows: paved areas in grey, green areas in green, and buildings in blue (AV Swiss Cadastral Surveying, 2016). The measuring sites are visible as red dots. IND: CSO Industry in Fehraltorf, OFH: Rumlikon and one-family houses in Fehraltorf, TWN: CSO Morgenthal in Russikon.

# BGI parameters

Implementation of BGI in the model: BC and PP were only implemented if the area exceeds 2 m^2^. A minimum area larger than 10 m^2^ was assumed for GR, while a UW area greater than 18 m^2^ was selected as a minimum requirement.

**Table SI 6.** Values used for the BGI parameters implemented in SWMM (Joshi et al., 2021; Leimgruber et al., 2018; Rodriguez et al., 2023).

| **Layer** | **Parameter** | **BC** | **PP** | **GR** | **UW** |
| --- | --- | --- | --- | --- | --- |
|  |  |  |  |  |  |
| Surface | Berm height [mm] | 150 | - | 50 | 2000 |
|  | Vegetation volume fraction | 0.1 | - | 0.5 | - |
|  | Roughness (Manning's n) [-] | 0.2 | 0.012 | 0.2 | - |
|  | Surface slope [%] | 1 | 1 | 1 | - |
| Soil/Sand | Thickness [mm] | 600 | - | 150 | - |
|  | Porosity (volume fraction) | 0.5 | - | 0.45 | - |
|  | Field capacity (volume fraction) | 0.2 | - | 0.2 | - |
|  | Wilting point (volume fraction) | 0.1 | - | 0.1 | - |
|  | Conductivity [mm/h] | 250 | - | 120 | - |
|  | Conductivity slope | 12.5 | - | 5 | - |
|  | Suction head [mm] | 50 | - | 49.5 | - |
| Pavement | Thickness [mm] |  | 150 | - | - |
|  | Void ratio (Voids/Solids) |  | 0.15 | - | - |
|  | Impervious surface fraction |  | 0 | - | - |
|  | Permeability [mm/h] |  | 500 | - | - |
| Drainage mat | Thickness [mm] |  | - | 75 | - |
|  | Void fraction |  | - | 0.75 | - |
|  | Roughness (Manning's n) [-] |  | - | 0.1 | - |
| Storage | Thickness/Height [mm] | 150 | 300 | - | - |
|  | Void ratio (Voids/Solids) | 0.75 | 0.4 | - | - |
|  | Seepage factor [mm/h] | 70 | 7 | - | - |
| Underdrain | Flow coefficient [mm/h] | 0.5 | 0.5 | - | 36 |
|  | Flow exponent | 0.5 | 0.5 | - | 0.5 |
|  | Offset height [mm] | 150 | 100 | - | 1500 |

References

Ahlers, J., Riedhammer, C., Vogliano, M., Ebert, R.U., Kühne, R. and Schüürmann, G. 2006. Acute to chronic ratios in aquatic toxicity—variation across trophic levels and relationship with chemical structure. Environmental Toxicology and Chemistry: An International Journal 25(11), 2937-2945.

AV Swiss Cadastral Surveying 2016. Amtliche Vermessung. <https://www.cadastre.ch>.

Brinkmann, M., Montgomery, D., Selinger, S., Miller, J.G., Stock, E., Alcaraz, A.J., Challis, J.K., Weber, L., Janz, D. and Hecker, M. 2022. Acute toxicity of the tire rubber-derived chemical 6PPD-quinone to four fishes of commercial, cultural, and ecological importance. Environmental Science & Technology Letters 9(4), 333-338.

Ecotoxcentre 2023. Proposals for Quality Criteria for Surface Waters. <https://www.oekotoxzentrum.ch/expertenservice/qualitaetskriterien/qualitaetskriterienvorschlaege-oekotoxzentrum/> (accessed: 07.08.2023).

Gujer, W. (2007) Siedlungswasserwirtschaft, Springer Berlin, Heidelberg. <https://doi.org/10.1007/978-3-540-34330-1>.

Hörnschemeyer, B., Henrichs, M., Dittmer, U. and Uhl, M. 2023. Parameterization for Modeling Blue–Green Infrastructures in Urban Settings Using SWMM-UrbanEVA. Water 15(15), 2840.

Joshi, P., Leitão, J.P., Maurer, M. and Bach, P.M. 2021. Not all SuDS are created equal: Impact of different approaches on combined sewer overflows. Water Research 191, 116780.

Leimgruber, J., Krebs, G., Camhy, D. and Muschalla, D. 2018. Sensitivity of model-based water balance to low impact development parameters. Water 10(12), 1838.

R Development Core Team 2010 R: A language and environment for statistical computing, R foundation for Statistical Computing.

Rodriguez, M., Cavadini, G.B. and Cook, L. 2023 The effect of model structure and assumptions on combined sewer overflows and green stormwater infrastructure.

Sandré, F., Huynh, N., Gromaire, M.-C., Varrault, G., Morin, C., Moilleron, R., Le Roux, J. and Garrigue-Antar, L. 2022. Road Runoff Characterization: Ecotoxicological Assessment Combined with (Non-) Target Screenings of Micropollutants for the Identification of Relevant Toxicants in the Dissolved Phase. Water 14(4), 511.

Slobodnik, J., Mrafkova, L., Carere, M., Ferrara, F., Pennelli, B., Schüürmann, G. and von der Ohe, P.C. 2012. Identification of river basin specific pollutants and derivation of environmental quality standards: A case study in the Slovak Republic. TrAC Trends in Analytical Chemistry 41, 133-145.

Spahr, S., Teixidó, M., Sedlak, D.L. and Luthy, R.G. 2020. Hydrophilic trace organic contaminants in urban stormwater: occurrence, toxicological relevance, and the need to enhance green stormwater infrastructure. Environmental Science: Water Research & Technology 6(1), 15-44.

Tamis, J.E., Koelmans, A.A., Dröge, R., Kaag, N.H., Keur, M.C., Tromp, P.C. and Jongbloed, R.H. 2021. Environmental risks of car tire microplastic particles and other road runoff pollutants. Microplastics and Nanoplastics 1(1), 1-17.

TGD, E. 2003. Technical guidance document on risk assessment in support of commission directive 93/67/EEC on risk assessment for new notified substances, Commission Regulation (EC) No 1488/94 on Risk Assessment for existing substances, and Directive 98/8/EC of the European Parliament and of the Council concerning the placing of biocidal products on the market. Part I–IV, European Chemicals Bureau (ECB), JRC-Ispra (VA), Italy.

Tian, Z., Zhao, H., Peter, K.T., Gonzalez, M., Wetzel, J., Wu, C., Hu, X., Prat, J., Mudrock, E., Hettinger, R., Cortina, A.E., Biswas, R.G., Kock, F.V.C., Soong, R., Jenne, A., Du, B., Hou, F., He, H., Lundeen, R., Gilbreath, A., Sutton, R., Scholz, N.L., Davis, J.W., Dodd, M.C., Simpson, A., McIntyre, J.K. and Kolodziej, E.P. 2021. A ubiquitous tire rubber–derived chemical induces acute mortality in coho salmon. Science 371(6525), 185-189.

UVEK (Eidgenössisches Departement für Umwelt, Verkehr, Energie und Kommunikation), 2020 Gewässerschutzverordnung (GSchV), Änderung vom 13. Februar 2020.

von der Ohe, P.C., Dulio, V., Slobodnik, J., De Deckere, E., Kühne, R., Ebert, R.-U., Ginebreda, A., De Cooman, W., Schüürmann, G. and Brack, W. 2011. A new risk assessment approach for the prioritization of 500 classical and emerging organic microcontaminants as potential river basin specific pollutants under the European Water Framework Directive. Science of the Total Environment 409(11), 2064-2077.
